# BI-RADS 3–5 microcalcifications: prediction of lymph node metastasis of breast cancer

**DOI:** 10.18632/oncotarget.16318

**Published:** 2017-03-17

**Authors:** Dongzhi Cen, Li Xu, Siwei Zhang, Shuqin Zhou, Yan Huang, Zhiguang Chen, Ningna Li, Yuan Wang, Qun Wang

**Affiliations:** ^1^ Department of Radiation Oncology and Department of Nuclear Medicine, The Third Affiliated Hospital of Guangzhou Medical University, Guangzhou, 510150, Guangdong, P.R. China; ^2^ Department of Radiology, The Second Affiliated Hospital of Guangzhou University of Chinese Medicine and Guangdong Provincial Hospital of Chinese Medicine, Guangzhou, Guangdong Province 510120, P.R. China; ^3^ Jishou University, Jishou Hunan 427200, P.R. China

**Keywords:** lymph node metastasis, mammography, calcification, infiltrating ductal carcinoma, logistic regression

## Abstract

**Purpose:**

To determine whether the clinicopathological parameters and Breast Imaging Reporting and Data System (BI-RADS) 3–5 microcalcifications differed between lymph node positive (LN (+)) and lymph node negative (LN (−)) invasive ductal carcinoma (IDC).

**Results:**

For microcalcification-associated breast cancers, seven selected features (age, tumor size, Ki-67 status, lymphovascular invasion, calcification range, calcification diameter and calcification density) were significantly associated with LN status (all *P* < 0.05). Multivariate logistic regression analysis found that three risk factors (age: older vs. younger OR: 0.973 *P* = 0.006, tumor size: larger vs. smaller OR: 1.671, *P* < 0.001 and calcification density: calcifications > 20/cm^2^ vs. calcifications ≤ 20/cm^2^ OR: 1.698, *P* < 0.001) were significant independent predictors. This model had an area under the receiver operating characteristic curve (AUC) of 0.701. The nodal staging (N0 and N1 *χ*^2^ = 5.701, *P* = 0.017; N0 and N2 *χ*^2^ = 6.614, *P* = 0.013) was significantly positively associated with calcification density. The luminal B subtype had the highest risk of LN metastasis. Multivariate analysis demonstrated that calcification > 2 cm in range (OR: 2.209) and larger tumor size (OR: 1.882) were independently predictive of LN metastasis in the luminal B subtype (AUC = 0.667).

**Materials and Methods:**

Mammographic images of 419 female breast cancer patients were included. Associations between the risk factors and LN status were evaluated using a Chi-square test, ANOVA and binary logistic regression analysis.

**Conclusions:**

This study found that age, tumor size and calcifications density can be conveniently used to facilitate the preoperative prediction of LN metastasis. The luminal B subtype has the highest risk of LN metastasis among the microcalcification-associated breast cancers.

## INTRODUCTION

Breast cancer is one of the most frequent malignancies worldwide and represents an important public health problem [1, 2]. Evaluating the status of axillary lymph nodes (ALNs) is essential in deciding appropriate treatment and staging as well as predicting the long-term survival in breast cancer [3]. Although significant progress has been made in the genetic and molecular characterization of breast malignant lesions, axillary lymph node involvement is the single most important prognostic variable [4–7].

Previous studies have used various factors to predict lymph node metastasis [8–11] such as magnetic resonance spectroscopy, DNA microarray assay for gene expression in breast cancer tissues, and P53 and Ki67 in patients with estrogen receptor (ER)-positive and human epidermal growth factor receptor 2 (HER2)-negative breast cancer [9, 12, 13].

The spread of screening mammography has led to increasing occurrences of microcalcifications [14, 15]. Mammographically detected microcalcifications represent the earliest mammographic findings of non-palpable breast cancers, which are found in approximately 70% of minimal breast carcinomas [16, 17] To the best of our knowledge, no studies have determined whether a calcification features combined with clinicopathological parameters would enable superior prediction of LN metastasis in IDC of breast. Therefore, we investigated whether the clinicopathological parameters and imaging features of the patterns of mammographically detected calcifications differed between LN (+) tumors and LN (−) tumors.

## RESULTS

**Figure 1:**
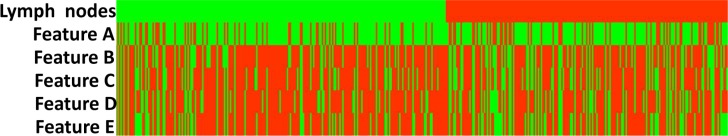
Clustering of samples of lymph node involvement (*N* = 419) Line1—LN (+)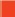, LN (−)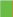. Features A–E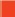 ①Fine linear/branching/pleomorphic ① Grouped or clustered or regional ① calcifications ≤ 2 cm in range ① califications ≤ 0.5 cm in diameter ① califications ≤ 20/cm^2^ in density. Features A–E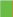 ②amorphour/coarse heterogenous ②line or segmental ②calcifications > 2 cm in range ②calcifications > 0.5 cm in diameter ②calcifications > 20/cm^2^ in density.

Hierarchical clustering displayed clear grouping of samples of LN involvement (Figure 1).

**Figure 2:**
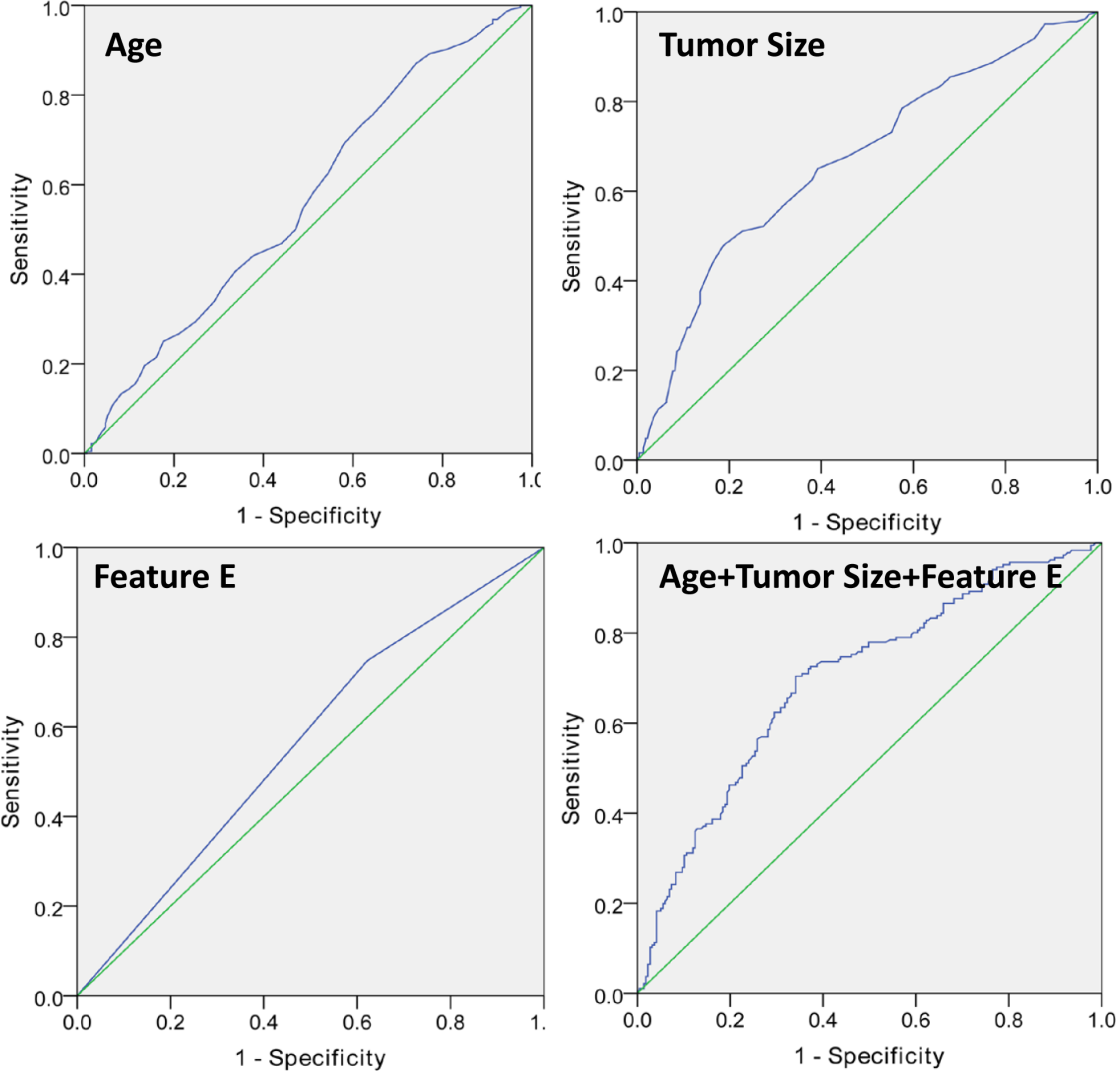
Invasive carcinomas associated with microcalcification (Feature E: calcifications with > 20/cm^2^ in density)

For microcalcification-associated breast cancers, seven selected features (age, tumor size, Ki-67 status, lymphovascular invasion, calcification range, calcification diameter and calcification density) were significantly associated with LN status (all *P* < 0.05). Multivariate logistic regression analysis showed that three risk factors (age: older vs. younger OR: 0.973 *P* = 0.006, tumor size: larger vs. smaller OR: 1.671, *P* < 0.001 and calcification density: calcifications > 20/cm^2^ vs. calcifications ≤ 20/cm^2^ OR: 1.698, *P* < 0.001) were significant independent predictors. This model had an area under the receiver operating characteristic curve (AUC) of 0.701. The nodal staging (N0 and N1 χ^2^ = 5.701, *P* = 0.017; N0 and N2 χ^2^ = 6.614, *P* = 0.013) was significantly positively associated with calcification density (Figure 2).

**Figure 3:**
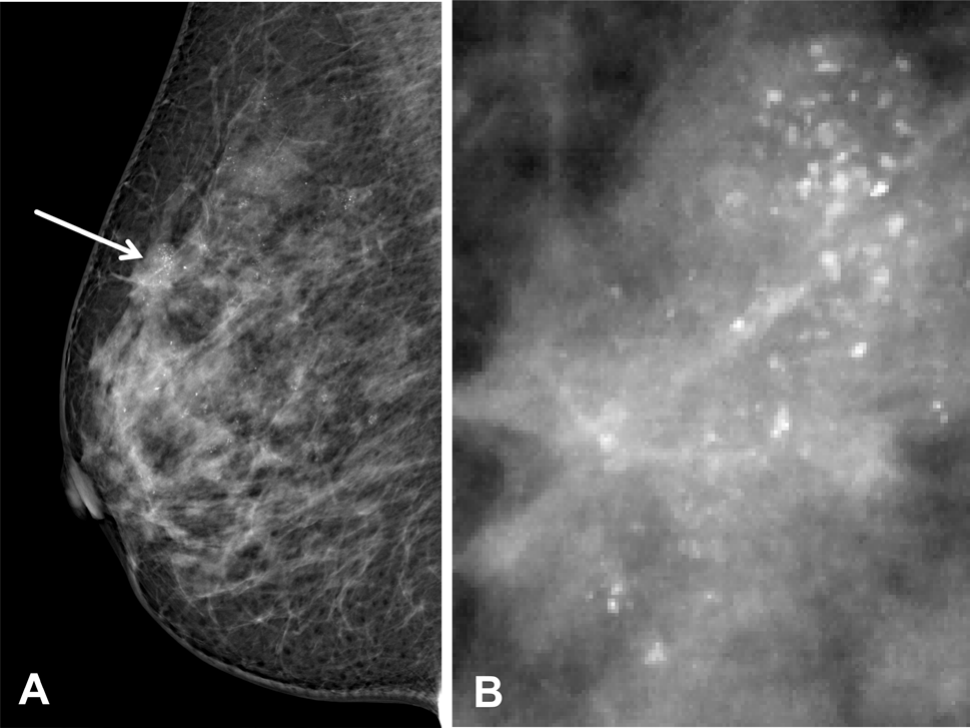
Three risk factors (age, tumor size and Feature E) were statistically significant independent predictors, And the area under the receiver operating characteristic curve for predicting LNM was 0.70

We demonstrated that larger tumor size, younger age and calcifications > 20/cm^2^ in density (Figure 3) were associated with a significantly higher incidence of LN metastasis (Tables 1, 2, 3). This model had an AUC of 0.701.

**Table 1:** Clinical and pathologic characteristics of 419 patients between LN(−) and LN(+) tumors

Characteristics	LN (−)	LN (+)	*P* value
Age, years, mean (range) (*N* = 418)	52.65 ± 10.82	50.31 ± 11.19	**0.031**
Tumor size (cm) (*N* = 405)	1.87 ± 1.04	2.48 ± 1.23	**0.000**
ER (*N* = 419)	52.03 ± 39.62	50.77 ± 38.96	0.745
PR (*N* = 419)	30.93 ± 36.28	31.27 ± 35.63	0.922
Ki-67 (*N* = 413)	25.49 ± 19.56	29.63 ± 21.36	**0.041**
ER (*N* = 419)			**0..844**
Negative	77 (34.1)	64 (33.2)	
Positive	149 (65.9)	129 (66.8)	
PR (*N* = 419)			**0.403**
Negative	117 (51.8)	92 (47.7)	
Positive	109 (48.2)	101 (52.3)	
Ki-67 (*N* = 413)			0.016
Negative	77 (34.5)	45 (23.7)	
Positive	146 (65.5)	145 (76.3)	
HER-2 (*N* = 346)			0.926
Negative	105 (55.6)	88 (56.1)	
Positive	84 (44.4)	69 (43.9)	
Histological grade (*N* = 418)			**0.130**
I	19 (8.4)	9 (4.7)	
II–III	207 (91.6)	183 (95.3)	
Lymphovascular invasion (*N* = 413)			0.000
Yes	31 (13.9)	86 (45.3)	
No	192 (86.1)	104 (54.7)	

**Table 2:** Comparison of features A-E between LN(−) and LN(+) tumors (*N* = 419)

	LN (−)(*n* = 226)	LN (+)(*n* = 226)	*P* value
**Feature A (Calcification morphology)**			0.123
Fine linear/branching/pleomorphic	55 (24.3)	60 (31.1)	
Amorphour/Coarse heterogenous	171 (75.7)	133 (68.9)	
**Feature B (Calcification distrubution)**			0.058
Grouped or Clustered or Regional	169 (74.8)	128 (66.3)	
Linear or Segmental	57 (25.2)	65 (33.7)	
**Feature C**			**0.019**
Calcifications ≤ 2 cm in range	170 (75.2)	125 (64.8)	
Calcifications > 2 cm in range	56 (24.8)	68 (35.2)	
**Feature D**			**0.047**
Calcifications ≤ 0.5 mm in diameter	172 (76.1)	130 (67.4)	
Calcifications > 0.5 mm in diameter	54 (23.9)	63 (32.6)	
**Feature E**			**0.005**
Calcifications ≤ 20/cm^2^ in density	169 (74.8)	120 (62.2)	
Calcifications > 20/ cm^2^ in density	57 (25.2)	73 (37.8)	

Note – Numbers in parentheses are percentages.

**Table 3:** Binary logistic regression analysis of prognostic factors for lymph node metastasis of breast cancer

	β	S.E.	Wald	Sig.	OR	95.0% C.I.for EXP (β)	
						Lower	Upper
Age	−0.028	0.01	7.62	0.006	0.973	0.954	0.992
Tumor size	0.514	0.107	23.068	0.000	1.671	1.355	2.061
Feature E	0.529	0.231	5.264	0.022	1.698	1.080	2.668
Constant	0.017	0.550	0.001	0.975	1.017		

Nodal staging (N0 and N1 χ^2^ = 5.701, *P* = 0.017; N0 and N2 χ^2^ = 6.614, *P* = 0.013) was significantly associated with Feature E (Tables 4, 5).

**Table 4:** Comparison of features A-E between different nodal staging (TNM stage *N* = 419)

	N0(*n* = 226)	N1(*n* = 114)	N2(*n* = 49)	N3(*n* = 30)	*P* value
**Feature E**					**0.027**
*Calcifications ≤ 20/cm^2^ in density*	169 (58.5)	71 (24.6)	28 (9.7)	21 (7.3)	
*Calcifications* > *20/cm^2^ in density*	57 (43.8)	43 (33.1)	21 (16.2)	9 (6.9)	

Note – Numbers in parentheses are percentages.

**Table 5:** Comparison of feature E between different nodal staging

		χ^2^	*P* value
N0	N1	5.701	**0.017**
	N2	6.164	**0.013**
	N3	0.316	0.574
N1	N2	0.379	0.538
	N3	0.613	0.434
N2	N3	1.306	0.253

Regarding the microcalcification-associated breast cancers, 68 (17.5%) were Luminal A, 197 (50.6%) were Luminal B, 94 (24.2%) were HER2 and 30 (7.7%) were basal subtypes (*N* = 389). We demonstrated that the Luminal B subtype (Luminal B vs. Luminal A, Luminal B vs. others, Luminal B vs. Basal) have the highest risk of LN metastasis (Table 1). Univariate analysis found that three features (tumor size, lymphovascular invasion and calcification range) were significantly associated with LN status of the Luminal B molecular subtype (all *P* < 0.05). Multivariate analysis showed that calcification > 2 cm in range (OR: 1.878 95%CI: 1.150 to 3.067) and tumor size (OR: 1.882 95%CI: 1.327 to 2.670) were independently predictive of LN metastasis of the Luminal B molecular subtype (Table 6). This model had an AUC of 0.667.

**Table 6:** Breast cancer molecular subtypes between LN(−) and LN(+) tumors

Characteristics	LN (−)	LN (+)	*P* value
Molecular subtypes (*N* = 389)			**0.013**
Luminal A	46 (21.8)	22 (12.4)	
Luminal B	93 (44.1)	104 (58.4)	
HER2	52 (24.6)	42 (23.6)	
Basal	20 (9.5)	10 (5.6)	
Luminal A vs. others (*N* = 389)			**0.015**
Yes	46 (21.8)	22 (12.4)	
No	165 (78.2)	156 (87.6)	
Luminal B vs. others (*N* = 389)			**0.005**
Yes	93 (44.1)	104 (58.4)	
No	118 (55.9)	74 (41.6)	
HER2 vs. others (*N* = 389)			0.810
Yes	52 (24.6)	42 (23.6)	
No	159 (75.4)	136 (76.4)	
Basal vs. others (*N* = 389)			0.155
Yes	20 (9.5)	10 (5.6)	
No	191 (90.5)	168 (94.4)	
Luminal A vs. Luminal B (*N* = 265)			**0.004**
Luminal A	46 (33.1)	22 (17.5)	
Luminal B	93 (66.9)	104 (82.5)	
Luminal A vs. HER2 (*N* = 162)			0.113
Luminal A	46 (46.9)	22 (34.4)	
HER2	52 (53.1)	42 (65.6)	
Luminal A vs. Basal (*N* = 98)			0.924
Luminal A	46 (69.7)	22 (68.8)	
Basal	20 (30.3)	10 (31.3)	
Luminal B vs. HER2 (*N* = 291)			0.196
Luminal B	93 (64.1)	104 (71.2)	
HER2	52 (35.9)	42 (28.8)	
Luminal B vs. Basal (*N* = 227)			0.047
Luminal B	93 (82.3)	104 (91.2)	
Basal	20 (17.7)	10 (8.8)	
HER2 vs. Basal (*N* = 124)			0.273
HER2	52 (72.2)	42 (80.8)	
Basal	20 (27.8)	10 (19.2)	

There were no significant differences in clinicopathological parameters or BI-RADS 3–5 microcalcifications between the LN (−) and LN (+) invasive ductal carcinoma (Luminal A, HER2, Basal molecular subtype).

## DISCUSSION

Mammographically detected calcifications are frequently used as the only sign of breast cancer [18]. Mammography is the gold standard modality for detecting microcalcifications [19]. BI-RADS 3–5 microcalcifications are a characteristic appearance of breast cancer at mammographic imaging and a well-known criterion in the diagnosis of the disease. LN metastasis is one of the most important prognostic factors in IDC patients. Patients with LN metastasis have an approximately four- to eight-fold higher mortality rate than those without nodal involvement [20]. To the best of our knowledge, no studies have determined whether calcification features combined with clinicopathological parameters would enable the prediction of LN metastasis.

Microcalcifications depicted on mammographic imaging develop in (i) luminal secretions or (ii) the necrotic cellular debris in the lumen of the distended ducts [21]. The microcalcifications that develop in necrotic cellular debris are irregular borders as well as linear with clefts in a focal, segmental or regional distribution [21]. However, the microcalcifications that develop in luminal secretions are round and punctate as well as amorphous calcifications within a cluster [21]. The characteristics of breast microcalcifications continue to attract interest. Hashimoto and coworkers found that patients with microcalcifications were significantly more likely to have LN metastases [19]. Li and coworkers found that malignant-appearing microcalcifications were significantly associated with a LN (+) status and that they always presented in breast cancer patients who were non-menopausal as well as with a family history of carcinoma [22]. Howland and coworkers reported that HER2 positivity is recognized to be associated with a higher incidence of LN metastases [23]. Several factors, including a higher ER-positivity rate, the prevalence of c-myc expression [24], as well as the elevated expression of osteopontin [25] and the ryanodine receptor 3 gene [26], were believed to contribute to the microcalcifications. Several factors, including HER2 positivity [23], the number of a CK19 mRNA copies [27], an elevated expression of osteopontin [25], T size and LVI [27], tumor grade [28], and clinical stage [28] were believed to contribute to lymph node metastases. However, questions regarding the most significant factor affecting lymph node metastases or the presence of microcalcifications remain unanswered.

If it is possible to predict the LN metastasis based on the patterns of mammographically detected calcifications, this information will be essential for clinical decision-making [29]. The multivariate analysis in our study demonstrates that the clinicopathological and imaging parameters of infiltrating ductal carcinoma, which consisted of three selected features (age, tumor size and Feature E), were statistically significant independent predictors. We demonstrated that larger tumor size, younger age and calcifications > 20/ cm^2^ were associated with a significantly higher rate of LN metastasis. However, other studies did not perform other measurements such as calcification range, calcification diameter and calcification density to more comprehensively evaluate the appearance of these calcifications. Additionally, our study is the first study to identify the risk factors in IDC, including a relatively large number of breast cancer patients. The discrimination of the model for predicting LNM was 0.70 in this study was 0.70 (95%, C.I. 0.69–0.73), thereby confirming a high level of reliability.

A previous study reported that HER2 positivity is associated with a higher rate of LN metastases [23]; this was not confirmed by our study. However, little is known about the incidence of microcalcification-associated breast cancers [15]. Our study found that Luminal B tumors (Luminal B vs. others, Luminal B vs. Luminal A, Luminal B vs. basal) have the highest risk of LN metastasis. Future data from large studies will be of interest. Our multivariate analysis showed that calcification > 2 cm in range (OR: 1.878 95% CI: 1.150 to 3.067) and tumor size (OR: 1.882 95% CI: 1.327 to 2.670) were independently predictive of LN metastasis of the Luminal B molecular subtype. The discrimination of the present study's model for predicting LNM was 0.67. And future data from large studies will be of interest.

Our study had limitations. First, we did not evaluate interobserver variability because this was a retrospective analysis and two radiologists reviewed the mammographic images in consensus. Secondly, we did not determine whether microcalcifications were combined with associated findings such as focal asymmetry, architectural distortion, or suspicious masses. Further study is needed to explore additional relationships. Thirdly, micrometastases were found in 22 (5.3%) of 419 patients. Due to the limitations of our raw data (micrometastases 5.3%), the clinicopathological parameters and BI-RADS 3–5 microcalcifications only predict positive lymph node status.

In conclusion, our findings clearly show that age, tumor size and Feature E (≤ 20 or > 20/cm^2^ in density) can be conveniently used to facilitate the preoperative individualized prediction of lymph node metastasis in patients with IDC. The discriminatory power for this prediction model was good with an overall AUC of 0.70. This information may be useful for clinical decision-making in breast cancer patients.

## MATERIALS AND METHODS

### Study subjects

The ethics committee approved the study (Guangdong Provincial Traditional Chinese Medicine Hospital), and written informed consent was obtained from all breast cancer patients. Patients were included in this analysis if information on (1) invasive ductal carcinoma of breast (2) Breast Imaging Reporting and Data System (BI-RADS) 3–5 microcalcifications and (3) histopathologic information were available.

Between January 2011 and April 2016, 419 female patients (aged 51.7 ± 10.8 years; range, 25–88 years) met the selection criteria and were included.

### Mammography interpretations

The digital mammograms acquired were analyzed using a standard four-view film. All cases of microcalcifications were classified according to the method proposed by the American College of Radiology, and only those classified as BI-RADS 3–5 were selected [16, 30, 31]. All of the parameters of the calcifications (Features A-E) were divided in a binary manner. We conducted a detailed image analysis to evaluate morphology (Feature A (1) Fine linear or branching or pleomorphic (2) amorphous or coarse heterogeneous), distribution (Feature B (1) grouped or clustered or regional (2) linear or segmental), range (Feature C (1) calcifications measuring ≤ 2 cm or (2) > 2 cm in range), diameter (Feature D (1) ≤ 0.5 mm or (2) > 0.5 mm in diameter) and density (Feature E (1) ≤ 20 or (2) > 20/cm^2^ in density).

### Histopathologic assessment

Histopathologic information, including the progesterone receptor status, histological grade, ER status, HER-2, Ki-67 (Ki67 ≤ 14% was defined as low expression and Ki67 > 14% as high expression [32–34]), tumor size, lymphovascular invasion (IVI) and LN status (number of ALND, ALND(+), number of SLNB, SLNB(+) and micrometastasis), were obtained from the pathology reports.

Tumors were divided into 4 molecular subtypes according to previous reports [32–34]: (1) the Luminal A subtype, (2) the Luminal B subtype, (3) the HER-2 enriched subtype, and (4) the Basal subtype.

HER2-positive status (IHC 3+ or Fish+ and IHC 0/1+ or Fish-) was defined by the 2013 American Society of Clinical Oncology/College of American Pathology guidelines in our study [25, 35].

**Figure 4:**
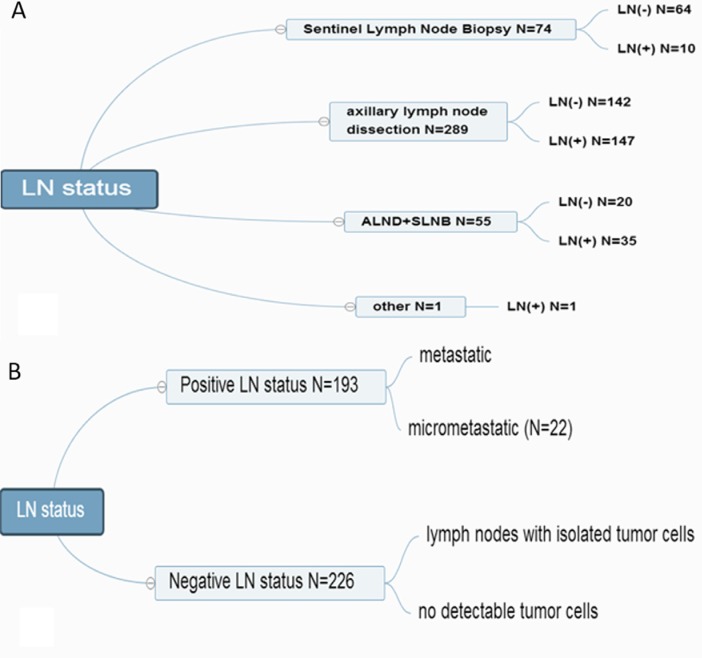
LN-positive and LN-negative status

LN was considered positive based on the HE staining and IHC test. Each node was classified as having (i) macrometastasis (>2.0 mm in size), (ii) micrometastasis (> 0.2–2.0 mm in size), (iii) isolated tumor cells (ITC < 0.2 mm in size), or (iv) no detectable tumor cells (the Seventh Edition of the American Joint Committee on Cancer Classification) [24]. LN (+) status was defined as having micrometastatic or metastatic LN tumors; LN (−) status was defined as LNs with ITC or no detectable tumor cells [36] (Figure 4).

### Statistical analysis

Associations between the clinicopathological parameters and the patterns of mammographically detected calcifications as well as LN status were evaluated. A univariate analysis of variables was carried out using a Chi-square test and one-way analysis of variance (ANOVA) with a *P value* of < 0.05 as the limit of statistical significance. The variables that obtained a *P value* < 0.1 with univariate analysis were subjected to multistep multivariate binary logistic regression (version 15.0; SPSS Company, Chicago, IL).

## References

[1] Tabar L, Vitak B, Chen TH, Yen AM, Cohen A, Tot T, Chiu SY, Chen SL, Fann JC, Rosell J, Fohlin H, Smith RA, Duffy SW (2011). Swedish two-county trial: impact of mammographic screening on breast cancer mortality during 3 decades. Radiology.

[2] Amouzou KS, Amakoutou K, Bakriga B, Abalo A, Dossim A (2016). Hand replantation: First experience in a sub-Saharan African country (Togo). Indian journal of plastic surgery.

[3] Ferlay J, Shin HR, Bray F, Forman D, Mathers C, Parkin DM (2010). Estimates of worldwide burden of cancer in 2008: GLOBOCAN 2008. International journal of cancer.

[4] Kim JY, Seo HB, Park S, Moon JI, Lee JW, Lee NK, Lee SW, Bae YT (2015). Early-stage invasive ductal carcinoma: Association of tumor apparent diffusion coefficient values with axillary lymph node metastasis. European journal of radiology.

[5] Saiz E1, Toonkel R, Poppiti RJ, Robinson MJ (1999). Infiltrating breast carcinoma smaller than 0.5 centimeters: is lymph node dissection necessary?. Cancer.

[6] Sui WF, Chen X, Peng ZK, Ye J, Wu JT (2016). The Diagnosis of Metastatic Axillary Lymph Nodes of Breast Cancer By Diffusion Weighted Imaging: a meta-analysis and systematic review. World journal of surgical oncology.

[7] Silverstein MJ, Skinner KA, Lomis TJ (2001). Predicting axillary nodal positivity in 2282 patients with breast carcinoma. World journal of surgery.

[8] Kim JY, Park HS, Kim S, Ryu J, Park S, Kim SI (2015). Prognostic Nomogram for Prediction of Axillary Pathologic Complete Response After Neoadjuvant Chemotherapy in Cytologically Proven Node-Positive Breast Cancer. Medicine.

[9] Yildiz R, Urkan M, Hancerliogullari O, Kilbas Z, Ozturk E, Mentes MO, Gorgulu S (2015). Comparison of five different popular scoring systems to predict nonsentinel lymph node status in patients with metastatic sentinel lymph nodes: a tertiary care center experience. Springer Plus.

[10] Ohara M, Matsuura K, Akimoto E, Noma M, Doi M, Nishizaka T, Kagawa N, Itamoto T (2016). Prognostic value of Ki67 and p53 in patients with estrogen receptor-positive and human epidermal growth factor receptor 2-negative breast cancer: Validation of the cut-off value of the Ki67 labeling index as a predictive factor. Molecular and clinical oncology.

[11] Qiu SQ, Zeng HC, Zhang F, Chen C, Huang WH, Pleijhuis RG, Wu JD, van Dam GM, Zhang GJ (2016). A nomogram to predict the probability of axillary lymph node metastasis in early breast cancer patients with positive axillary ultrasound. Scientific reports.

[12] Sorensen KP, Thomassen M, Tan Q, Bak M, Cold S, Burton M, Larsen MJ, Kruse TA (2015). Long non-coding RNA expression profiles predict metastasis in lymph node-negative breast cancer independently of traditional prognostic markers. Breast cancer research.

[13] Bathen TF, Jensen LR, Sitter B, Fjosne HE, Halgunset J, Axelson DE, Gribbestad IS, Lundgren S (2007). MR-determined metabolic phenotype of breast cancer in prediction of lymphatic spread, grade, and hormone status. Breast cancer research and treatment.

[14] Nakauchi C, Naoi Y, Shimazu K, Tsunashima R, Nishio M, Maruyama N, Shimomura A, Kagara N, Shimoda M, Kim SJ, Noguchi S (2014). Development of a prediction model for lymph node metastasis in luminal A subtype breast cancer: the possibility to omit sentinel lymph node biopsy. Cancer letters.

[15] Rominger MB, Steinmetz C, Westerman R, Ramaswamy A, Albert US (2015). Microcalcification-Associated Breast Cancer: Presentation, Successful First Excision, Long-Term Recurrence and Survival Rate. Breast care.

[16] Rauch GM, Hobbs BP, Kuerer HM, Scoggins ME, Benveniste AP, Park YM, Caudle AS, Fox PS, Smith BD, Adrada BE, Krishnamurthy S, Yang WT (2016). Microcalcifications in 1657 Patients with Pure Ductal Carcinoma in Situ of the Breast: Correlation with Clinical, Histopathologic, Biologic Features, and Local Recurrence. Annals of surgical oncology.

[17] Li E, Li J, Song Y, Xue M, Zhou C (2014). A comparative study of the diagnostic value of contrast-enhanced breast MR imaging and mammography on patients with BI-RADS 3-5 microcalcifications. PloS one.

[18] Bassett LW (1992). Mammographic analysis of calcifications. Radiologic clinics of North America.

[19] Tot T, Tabar L (2011). The role of radiological-pathological correlation in diagnosing early breast cancer: the pathologist’s perspective. Virchows Archiv.

[20] Bogner W, Gruber S, Pinker K, Grabner G, Stadlbauer A, Weber M, Moser E, Helbich TH, Trattnig S (2009). Diffusion-weighted MR for differentiation of breast lesions at 3.0 T: how does selection of diffusion protocols affect diagnosis?. Radiology.

[21] Bae MS, Moon WK, Chang JM, Cho N, Park SY, Won JK, Jeon YK, Moon HG, Han W, Park IA (2013). Mammographic features of calcifications in DCIS: correlation with oestrogen receptor and human epidermal growth factor receptor 2 status. European radiology.

[22] Li JN, Xu J, Wang J, Qing C, Zhao YM, Liu PF (2014). Correlation between mammograghic findings and clinical/ pathologic features in women with small invasive breast carcinomas. Asian Pacific journal of cancer prevention.

[23] Howland NK, Driver TD, Sedrak MP, Wen X, Dong W, Hatch S, Eltorky MA, Chao C (2013). Lymph node involvement in immunohistochemistry-based molecular classifications of breast cancer. The Journal of surgical research.

[24] Mazzini RC, Elias S, Nazario AC, Kemp C, Logullo AF (2009). Prevalence of c-myc expression in breast lesions associated with microcalcifications detected by routine mammography. Sao Paulo medical journal.

[25] Wang X, Chao L, Ma G, Chen L, Jin G, Hua M, Liu H, Ouyang A, Zhang X (2010). Primary breast carcinoma: association of mammographic calcifications with osteopontin expression. Radiology.

[26] Zhang L, Liu Y, Song F, Zheng H, Hu L, Lu H, Liu P, Hao X, Zhang W, Chen K (2011). Functional SNP in the microRNA-367 binding site in the 3′UTR of the calcium channel ryanodine receptor gene 3 (RYR3) affects breast cancer risk and calcification. Proceedings of the National Academy of Sciences of the United States of America.

[27] Di Filippo F, Giannarelli D, Bouteille C, Bernet L, Cano R, Cunnick G, Sapino A (2015). Elaboration of a nomogram to predict non sentinel node status in breast cancer patients with positive sentinel node, intra-operatively assessed with one step nucleic acid amplification method. Journal of experimental & clinical cancer research.

[28] Chakraborty A, Bose CK, Basak J, Sen AN, Mishra R, Mukhopadhyay A (2016). Determinants of lymph node status in women with breast cancer: A hospital based study from eastern India. The Indian journal of medical research.

[29] Radenkovic S, Konjevic G, Isakovic A, Stevanovic P, Gopcevic K, Jurisic V (2014). HER2-positive breast cancer patients: correlation between mammographic and pathological findings. Radiation protection dosimetry.

[30] Stomper PC, Margolin FR (1994). Ductal carcinoma in situ: the mammographer’s perspective. American journal of roentgenology.

[31] Lee HJ, Kim EK, Kim MJ, Youk JH, Lee JY, Kang DR, Oh KK (2008). Observer variability of Breast Imaging Reporting and Data System (BI-RADS) for breast ultrasound. European journal of radiology.

[32] Radenkovic S, Milosevic Z, Konjevic G, Karadzic K, Rovcanin B, Buta M, Gopcevic K, Jurisic V (2013). Lactate dehydrogenase, catalase, and superoxide dismutase in tumor tissue of breast cancer patients in respect to mammographic findings. Cell biochemistry and biophysics.

[33] Metzger-Filho O, Tutt A, de Azambuja E, Saini KS, Viale G, Loi S, Bradbury I, Bliss JM, Azim HA, Ellis P, Di Leo A, Baselga J, Sotiriou C (2012). Dissecting the heterogeneity of triple-negative breast cancer. Journal of clinical oncology.

[34] Perou CM, Sorlie T, Eisen MB, van de Rijn M, Jeffrey SS, Rees CA, Pollack JR, Ross DT, Johnsen H, Akslen LA, Fluge O, Pergamenschikov A, Williams C (2000). Molecular portraits of human breast tumours. Nature.

[35] Liu S, Wu XD, Xu WJ, Lin Q, Liu XJ, Li Y (2016). Is There a Correlation between the Presence of a Spiculated Mass on Mammogram and Luminal A Subtype Breast Cancer?. Korean journal of radiology.

[36] Wolff AC, Hammond ME, Hicks DG, Dowsett M, McShane LM, Allison KH, Allred DC, Bartlett JM, Bilous M, Fitzgibbons P, Hanna W, Jenkins RB, Mangu PB (2013). Recommendations for human epidermal growth factor receptor 2 testing in breast cancer: American Society of Clinical Oncology/College of American Pathologists clinical practice guideline update. Journal of clinical oncology.

